# The effects of pulsed electromagnetic field therapy on muscle strength and pain in patients with end-stage knee osteoarthritis: a randomized controlled trial

**DOI:** 10.3389/fmed.2024.1435277

**Published:** 2024-10-16

**Authors:** Qian-wen Wang, Michael Tim-yun Ong, Gene Chi-wai Man, Alfredo Franco-Obregón, Ben Chi-yin Choi, Pauline Po-yee Lui, Daniel T. P. Fong, Ji-hong Qiu, Xin He, Jonathan Patrick Ng, Patrick Shu-hang Yung

**Affiliations:** ^1^Department of Orthopaedics and Traumatology, Faculty of Medicine, The Chinese University of Hong Kong, Prince of Wales Hospital, Hong Kong, Hong Kong SAR, China; ^2^Department of Surgery, Yong Loo Lin School of Medicine, National University of Singapore, Singapore, Singapore; ^3^School of Sport, Exercise and Health Sciences, Loughborough University, Loughborough, United Kingdom; ^4^School of Exercise and Health, Shanghai University of Sport, Shanghai, China; ^5^Department of Orthopaedics and Traumatology, Prince of Wales Hospital, Hong Kong, Hong Kong SAR, China

**Keywords:** PEMF therapy, knee extensor muscle strength, knee flexor muscle strength, pain, end-stage knee osteoarthritis

## Abstract

**Background:**

Osteoarthritis (OA) of the knee is one of the most common chronic degenerative joint conditions affecting aging population.

**Aim:**

To investigate the effectiveness of a combination of home-based exercise and pulsed electromagnetic field (PEMF) therapy to improve muscle strength, physical function, and pain.

**Methods:**

Sixty patients were randomly assigned to either home-based exercise alone (control group; *n* = 30) or combined with PEMF therapy (treatment group; *n* = 30) twice a week for eight weeks. Knee extension, flexion muscle strength, gait speed (GS), 5 time sit-to-stand test (5STS), Visual Analogue Scale (VAS) pain and Knee Injury and Osteoarthritis Outcome Score (KOOS) were recorded at baseline and 4 and 8 weeks.

**Results:**

Significant improvements in symptomatic knee extension muscle strength (SKE, *p* = 0.001), flexion strength (SKF, *p* = 0.011), contralateral knee extension muscle strength (CKE, *p* = 0.002), and flexion strength (CKF, *p* = 0.009) were observed for the PEMF treatment group at 8 weeks. Significant reductions in VAS pain scores were observed in both the treatment (*p* < 0.001, partial η^2^ = 0.505) and control (*p* < 0.001, partial η^2^ = 0.268) groups. Significant differences were reported between groups in the 4 (*p* = 0.010, partial η^2^ = 0.111) and 8 (*p* = 0.046, partial η^2^ = 0.068) week assessment in VAS pain. A significant time difference was found in GS and 5STS between baseline and week 8 (GS: difference 0.051, *p* = 0.026; 5STS: difference 2.327, *p* < 0.001) in the treatment group. The significant group difference at week 8 was observed in SKE (*p* = 0.013) in female patients while pain in male patients (*p* = 0.026). Patients aged over 70 years have a significantly superior improvement in SKE, SKF, and CKF after 8 weeks of PEMF therapy.

**Conclusion:**

The combination of PEMF therapy and home-based exercise superiorly improved knee muscle strength and reduced pain in end-stage knee OA subjects and showed a promising tendency to improve performance-based physical function. PEMF therapy was shown to preferentially benefit knee muscle strength in female patients and patients aged over 70 years, whereas male patients were more responsive to PEMF therapy in the form of pain relief.

**Clinical trial registration:**

clinicalTrials.gov, NCT05550428.

## Introduction

Osteoarthritis (OA) of the knee is one of the most common chronic degenerative joint conditions inflicting the progressively aging global population, being ranked as the 12th leading cause of disability worldwide (1). Knee OA results in muscle weakness, loss of physical function, and poor quality of life. Pain and disability worsen during the end-stage of the condition (2). In hopes of managing their condition, knee OA patients assume a substantial economic burden ranging between $12,400 and $16,000 in direct and indirect costs during their lifetimes (3). In Hong Kong, incurred incremental costs of €1,320, €4,377, and €19,715 are common per patient exhibiting mild OA, severe OA, or requiring joint replacement, respectively (4).

Knee-extensor muscle weakness is a known risk factor for the development (5) and progression of OA (6) as well as one of the strongest predictors of functional limitations in patients with knee OA (7). Compared to healthy, age-matched volunteers, end-stage knee OA patients commonly exhibit a 35% reduction in knee-extensor strength while awaiting total knee replacement (TKR) (8). OA knee patients having undergone TKR, on average, lose an additional 80% of their preoperative knee-extensor strength following the operation. Finally, preoperative knee-extensor strength is predictive of postoperative functional recovery following TKR surgery (9). Collectively, the available literature highlights the importance of maintaining preoperative knee-extensor strength for end-stage knee OA patients awaiting TKR and suggests that interventions capable of improving knee-extensor strength may increase functional mobility after TKR and, in a subset of cases, may ultimately postpone the need for surgery. Another study has indicated that both quadriceps and hamstring muscle strength were associated with performance-and self-reported physical function in knee OA patients (10).

Rehabilitation programs that target muscle strength through exercise have been found essential in managing knee OA. According to one systematic review and meta-analysis, a 30–40% increase in knee-extensor strength is associated with improved knee pain and disability (11). However, as moderate to high-intensity exercise is generally required for improving muscle strength and endurance in healthy adults (12), neural sensitization and pain may limit the ability of knee OA patients to undertake such levels of exercise (13), undermining their potential recovery.

The clinical use of PEMF therapies in orthopedics has been approved for over 40 years and commonly entail analgesic benefits (14, 15). When specifically targeting the knee, however, evidence that the technology improves pain, physical function, and quality of life has been inconclusive (16–19), probably due to the unaddressed muscle weakness. Therefore, the approach employed in the present study was to target the leg musculature for its delivery of regenerative agents that naturally promote healing (20, 21). The therapy entails a muscle-targeted, low-energy (1mT, 10 min/week) PEMF therapy previously used in human trials (22, 23). Notably, this same PEMF paradigm was shown to improve functional mobility, increase lean muscle mass, and reduce pain in a community cohort containing frail elderly subjects (23). This PEMF paradigm has been demonstrated effective in promoting human muscle regeneration by inducing mitochondrial adaptations (21, 24), similar to those invoked during oxidative muscle development in response to exercise (24, 25).

A clinical priority of the sports medicine field is to conceive achievable and cost-effective exercise programs for patients with end-stage knee OA due to its high prevalence and economic burden. Currently, TKR remains the gold standard for patients suffering from end-stage knee OA. Unfortunately, due to high demand and limited resources in Hong Kong, the waiting time for surgery is an astoundingly 3 to 9 years (26). This double-blinded randomized control trial aimed to investigate the efficacy of combining home-based exercise with a patented PEMF-based therapy to improve knee muscle strength, physical function and reduce pain in patients with end-stage OA knee.

## Materials and methods

### Study design

This study was a double-blinded, randomized control trial. All procedures performed in studies involving human participants were performed following the ethical standards of The Joint Chinese University of Hong Kong—New Territories East Cluster Clinical Research Ethics Committee and were approved by the committee under reference number 2022.185 and registered on ClinicalTrials.gov (number: NCT05550428). This study has complied with the Declaration of Helsinki and its later amendments or comparable ethical standards. All the participants have given their oral and written consent.

### Participants

Participants were recruited from the Prince of Wales Hospital, Hong Kong, from December 2022 to July 2023. A total of 135 patients with OA symptoms of the knee were screened for study eligibility. Sixty patients were recruited according to the following criteria. Inclusion criteria: (a) diagnosis of end-stage symptomatic knee OA (Kellgren-Lawrence classification Grade III-IV), (b) enlistment on a TKR surgical waiting list; (c) ability to comply with the trial assessments and fully comprehend questionnaires; (d) be over 50 years of age and; (e) ability to walk unaided over 6 meters. Exclusion criteria: (a) history of knee or hip surgeries; (b) body mass index over 30 kg/m^2^ and; (c) history of cancer.

### Definition of radiographically confirmed knee OA

Anteroposterior weight-bearing views were obtained from bilateral X-ray knee assessments while participants extended both knees. Two experienced orthopedists, blinded to the clinical data, evaluated the radiographic images according to the Kellgren-Lawrence (KL) grading system. Radiographically confirmed knee OA was defined as KL grades ≥2 for both knee (27). For the present study, we described end-stage OA as KL grades ≥3 in one or both knees.

### Interventions

The intervention group received active PEMF therapy plus home-based exercise, and the control group received sham PEMF therapy plus home-based exercise. PEMF therapy dosage was set as 10 min/session, two sessions/week for eight weeks.

### Home-based exercise

Senior hospital physiotherapists and orthopedists designed the home-based exercise based on published literature and current practice. Home-based exercise involved stretching of the hip, knee, and calf muscles in combination with strengthening exercises including seated isometric quadriceps muscle exercise (SQM), Seated heel slides exercise (SHL), Seated banded knee flexion (SBKF), Seated banded quadriceps muscle exercise (SBQ), and Semi-squat exercise (SS), the details can be seen in Table 1. All the participants were instructed on the proper performance of the exercise regimen by a senior physiotherapist before the commencement of the intervention. Subjects were then instructed to perform the home-based exercises on their own twice a week for eight weeks. Patients were requested to keep personal diaries detailing their completion of home-based exercise regimens. One dedicated research assistant kept a weekly record of the participant’s physical activity and their adherence to home-based exercise when they came to our lab for PEMF therapy.

**Table 1:** Prescribed home-based exercise for both groups.

Exercise	Procedure	Protocol
SQM	Patients was in a sitting position with both hands holding the chair. They were instructed to maximally activate their thigh muscles in order to straighten their knee and hold the contraction for 5 s.	Frequency 2 times/week Intensity 2 sets of 12 repetitions/once a time Progression 1–4th week: 2 sets of 12 repetitions 4–8th week: 3 sets of 12 repetitions
SHS	Patients was in a sitting position with their feet flat on the floor. They were instructed to bend knees as slowly as possible and then slowly straighten them, making sure feet stay on the ground.	
Seated knee flexion (SBKF)	Patients were in a sitting position. Put elastic bands attached around the ankle, the anchor opposite end in front of the patient. They were instructed to perform a maximum isometric hamstring contraction prior to the lifting phase of the exercise.	
Seated banded quadriceps (SBQ)	Put elastic bands around both ankles, with feet shoulder-width apart, and hands on the side of the chair. One foot against the elastic band, try to straighten the knee, the other foot on the ground to resist the elasticity.	
SS	Patients were asked to stand with hands on the back of the chair, and performed semi-squat to −45 degrees flexion at knees and held this position for 5 s.	

### PEMF exposure

**Figure 1:**
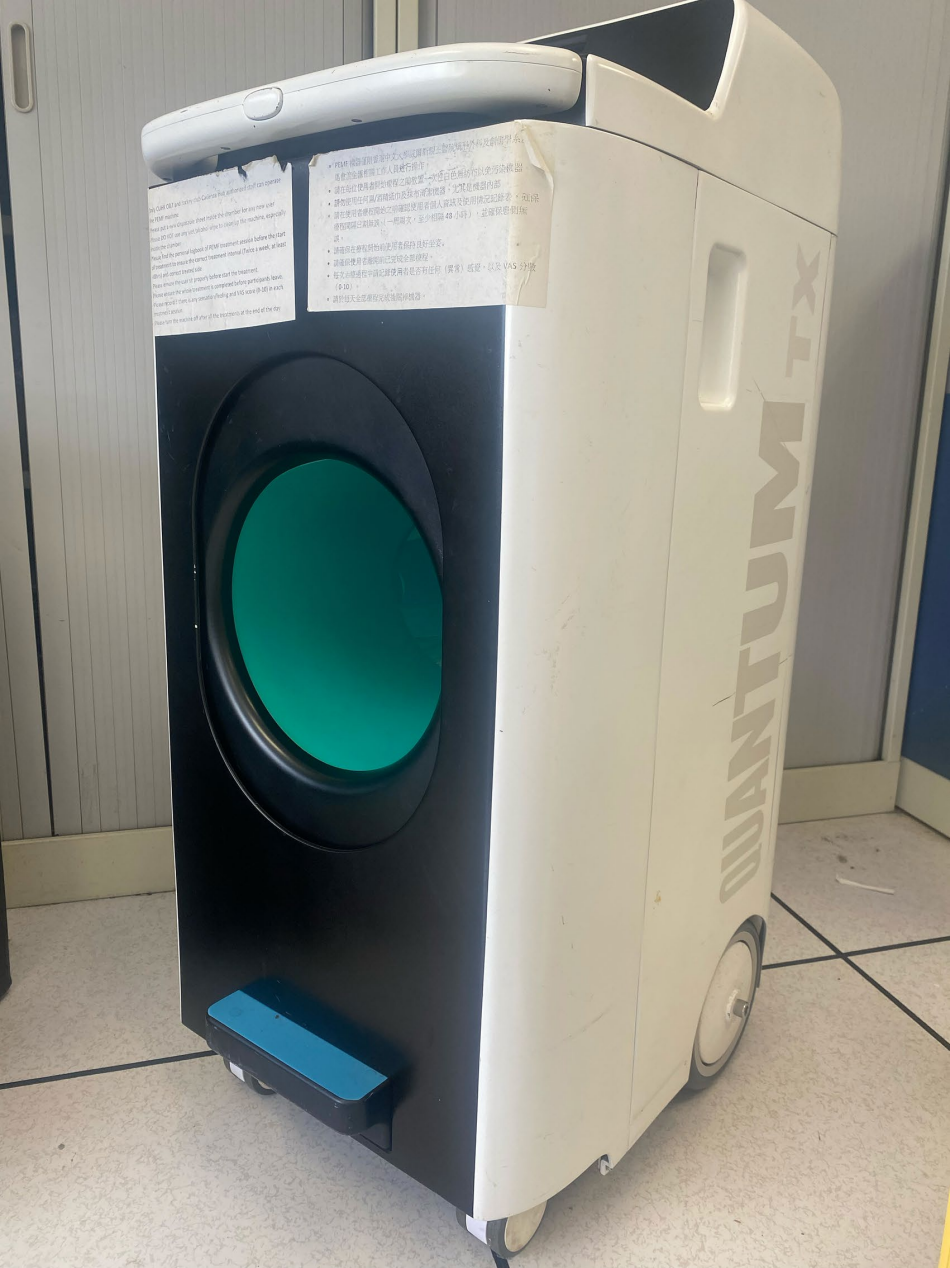
The PEMF device.

The quadriceps muscles of the upper leg and knees of participants were placed within the solenoids of the PEMF device (Figure 1). Alternating legs on consecutive visits were exposed to either PEMF or sham therapy for eight weeks for a total of 16 PEMF or sham sessions per participant, each leg receiving 8 exposures. The device is automatically designed to deliver uniform 1mT amplitude PEMFs at a frequency of 50 Hz for a duration of 10 min per session.

### Measurements

The primary outcome was knee muscle strength, and the secondary outcome was pain. Two testing assessors were blinded to the grouping allocations and were responsible for administrating the assessments at three time points: baseline (before the intervention), week 4 (end of week 4), and week 8 (end of week 8).

#### Knee muscle strength

A hand-held dynamometer (Hoggan Scientific, Salt Lake City, UT, USA) was used to assess knee extension and flexion muscle strength. The symptomatic side was defined as the one with the more severe OA knee according to radiographic features (Kellgren–Lawrence scale ≥3) as well as self-reported as the more painful of the two knees using the Visual Analogue Scale (VAS). The contralateral knee presented with the same radiographic diagnosis but was self-reported as the less painful of the two knees.

Participants were seated on an examination table with knees flexed at 60° from the plane of the table and feet off the ground. A hand-held dynamometer was positioned on the anterior aspect of the distal tibia, just superior to the malleoli, to measure maximal voluntary quadriceps muscle strength. To measure maximal voluntary isometric hamstring muscle strength the dynamometer was placed on the posterior part of the lower leg next to the malleoli. Participants grasped the examination table with both hands for stabilization and were instructed to extend or flex their knee “as hard as possible” against the hand-held dynamometer. Participants continued to exert force into the hand-held dynamometer for 5 s, and the maximum force across the trial was recorded. Maximal voluntary isometric contractions were repeated twice with the greatest value recorded. The peak force (N/kg) normalized by body mass was recorded for subsequent analysis.

#### 6-meter gait speed test (GS)

The six-meter gait speed test was used to measure the mean walking speed (in seconds) after 3 attempts of walking for 6 m along a straight line.

#### 5 time sit-to-stand test (5STS)

The 5 time sit-to-stand test is a physical performance test commonly-used in clinical geriatric studies (28, 29). Participants rose from a chair and returned to the seated position as quickly as possible for 5 repetitions, with arms were folded across their chests during the test. This test is easy to perform in clinical practice and has shown excellent intra- and inter-rater reliability (ICC, 0.89) in patients with severe hip or knee OA (30). Two trials were performed with approximately one-minute rest in between each trial as needed. Best performance of the two trials was computed for use in the analysis.

#### Self-reported pain

Self-report knee pain was assessed in response to the questions: (1) ‘How much pain have you had in the past week in your most painful knee? and (2) Could you mark it on this scale?’ Subjects were instructed to enter their responses on paper using a numerical rating scale (Visual Analogue Scale), where 0 indicated no pain and 10 the worst imaginable pain. Pain assessments were made at baseline, week 4 and week 8.

#### Knee injury and osteoarthritis outcome score (KOOS)

The KOOS collects data on five knee-specific patient-centered outcomes: Symptoms (seven items); Pain (nine items); Function in daily living (ADL, 17 items); Sport and Recreation Function (Sport/Rec, five items); and Quality of Life (QoL, four items). A Likert scale is used; all items have five possible answer options scored from 0 (No Problems) to 4 (Extreme Problems), and each of the five scores is calculated as the sum of the items included. Scores are transformed to a 0–100 scale, with zero representing extreme knee problems and 100 representing no knee problems, as common in orthopedic assessment scales and generic measures. 8–10 points may represent the minimal perceptible clinical improvement (MPCI) of the KOOS (31). The reliability and construct validity of KOOS has been extensively demonstrated in recent years (32). The KOOS-ADL subscale was used as self-reported physical function.

### Randomization and blinding

Participants were divided into two blocks of 10 persons per group, corresponding to PEMF intervention or sham groups. The PEMF intervention was administered using a commercial device (Quantum Tx, Singapore) as previously described (23). The delivery of PEMFs to the leg did not produce heat or cause any sensation, which allowed the participants to be blinded to the treatment. The PEMF device was activated by a personalized RFID card coded to deliver either the active or the sham treatment. The study participants used the RFID card to initiate the given treatment regime without their knowledge if the device delivered active or sham treatments. Outcome assessors were also blinded to the treatment group allocations.

### Data analysis

#### Sample size calculation

Quadriceps muscle strength was used as primary outcome for sample size estimation. The moderate effect size of the isometric quadriceps muscle strength was determined as 0.44 in patients with knee OA (33). A sample size calculation was performed for an analysis of covariance (ANCOVA) designed to detect significant differences in primary outcome between two groups with one covariate (baseline values). To ensure an 80% power such that a treatment effect could be detected at a two-sided 5% level of significance, a sample size of 30 participants per group was necessary, assuming a 20% dropout rate or other complications.

### Statistical analysis

The intention-to-treat (ITT) approach with the last-value-forward method was applied in this study. The results are expressed as means, standard deviations, and 95% confidence intervals (CIs). The significance level was set at *p* < 0.05. All data were analyzed using SPSS Version 29.0 (IBM, Chicago, IL).

A one-way repeated measure analysis of variance (ANOVA) was applied to each group to detect within-group differences in each outcome parameter among the three time points (i.e., baseline, week 4, week 8). *Post hoc* analysis with Bonferroni correction was conducted to explore the differences between the baseline and week 4, baseline and week 8, and week 4 and week 8.

A one-way analysis of covariance (ANCOVA) analysis with the baseline value as a covariate was analyzed at the week 4 assessment and week 8 assessment to determine the between-group differences on each parameter.

The effect size was calculated by partial eta-squared (η^2^). Partial η^2^ ≥ 0.01 was interpreted as the small effect, ≥ 0.06 as the medium effect, and ≥ 0.14 as the large effect. Group differences in baseline demographic and clinical variables were analyzed using independent t-tests for continuous data and Chi-square tests for categorical data.

Subgroup analysis was used to compare the outcomes across different gender and age groups via ANCOVA and independent t-test.

## Results

### Participants flow

**Figure 2:**
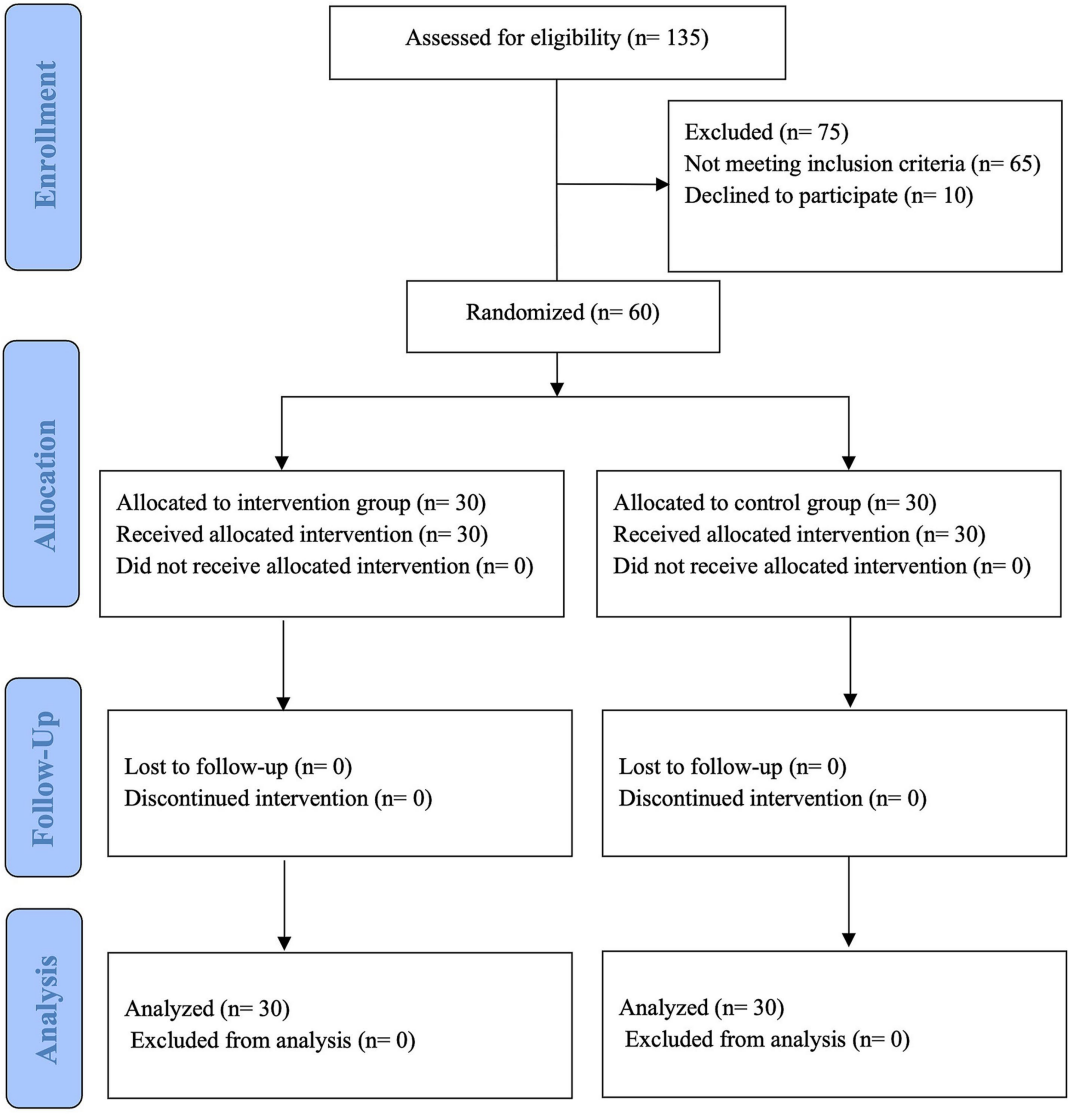
CONSORT flow diagram.

An initial pool of 135 older adults was screened for knee OA eligibility at the Prince of Wales Hospital, Hong Kong, from December 2022 to July 2023. Sixty qualified participants who signed the consent form were randomly allocated to either the treatment group (*n* = 30) or the control group (*n* = 30). No participants dropped out of the intervention. The participant flow chart according to the guideline of CONSORT is given in Figure 2.

### Baseline data

The demographic and clinical characteristics of the participants in each group are presented in Table 2. Most of the participants were female (66.7%) and had a long history of suffering from knee OA. The treatment group had a significantly higher body mass index (mean = 26.0) than the control group (mean = 24.1).

**Table 2:** Demographic and clinical characteristics at baseline.

	Treatment group (*n* = 30)	Control group (*n* = 30)	*p* value
Age (years)	69.8 ± 6.0	72.0 ± 5.6	0.135
*n*	30	30	/
Gender			0.100^a^
Male (%)	13 (43.3)	7 (23.3)	/
Female (%)	17 (56.7)	23 (76.7)	/
Height (cm)	157.5 ± 8.2	155 ± 9.1	0.451
Weight (kg)	64.0 ± 6.7	58.1 ± 9.8	0.011
BMI (kg/m^2^)	26.0 ± 4.0	24.1 ± 3.0	0.008
Durations of symptom (years)	10.4 ± 6.7	9.4 ± 6.0	0.539
Waiting time on the TKR list (years)	3.1 ± 1.6	3.4 ± 1.6	0.469
Symptomatic knee extension muscle strength (SKE) (N/kg)	2.40 ± 0.72	2.48 ± 0.91	0.686
Symptomatic knee flexion muscle strength (SKF) (N/kg)	1.86 ± 0.62	1.82 ± 0.69	0.822
Contralateral knee extension muscle strength (CKE) (N/kg)	2.72 ± 0.79	2.76 ± 0.92	0.864
Contralateral knee flexion muscle strength (CKF) (N/kg)	1.91 ± 0.58	1.87 ± 0.68	0.828
6-meter gait speed (m/s)	0.90 ± 0.24	0.86 ± 0.30	0.603
5-time Chair Stand Test (s)	13.1 ± 4.2	15.9 ± 6.5	0.057
VAS pain (cm)	5.93 ± 1.39	5.63 ± 1.60	0.442
KOOS- ADL (score)	71.2 ± 12.7	65.4 ± 17.4	0.141

Date expressed as Mean ± Standard Deviation. BMI, Body mass index; ^a^was analyzed using the Chi-square test.

### Within-group differences in knee muscle strength and pain

The one-way repeated measure ANOVA was conducted to compare the within-group effects. For the PEMF treatment group, a significant time difference was observed in symptomatic knee extension muscle strength (SKE) [*F*(2, 28) =7.059, *p* = 0.003, partial η^2^ = 0.335]. *Post hoc* analysis revealed a significant increase in SKE between baseline and week 8 assessments (difference = 0.459, 95% CI = 0.145–0.772, *p* = 0.003) and between week 4 and week 8 assessments (difference = 0.396, 95% CI = 0.050–0.746, *p* = 0.021).

A significant time difference was observed within the PEMF treatment group in symptomatic knee flexion muscle strength (SKF) [*F*(2, 28) =4.903, *p* = 0.015, partial η^2^ = 0.259]. *Post hoc* analysis revealed significant increases in SKF between baseline and 8 week assessments (difference = 0.292, 95% CI = 0.003–0.581, *p* = 0.047) and between 4 week and 8 week assessments (difference = 0.281, 95% CI = 0.037–0.525, *p* = 0.020).

Significant time differences were also observed in the PEMF treatment group in contralateral knee extension muscle strength (CKE) [*F*(2, 28) = 8.216, *p* = 0.002, partial η^2^ = 0.370] and knee flexion muscle strength (CKF) [*F*(2, 28) = 3.917, *p* = 0.032, partial η^2^ = 0.219]. *Post hoc* analysis revealed a significant increase in CKE between baseline and week 8 assessments (difference = 0.385, 95% CI = 0.095–0.675, *p* = 0.006) and between week 4 and week 8 assessments (difference = 0.422, 95% CI = 0.111–0.732, *p* = 0.005). There was also a significant increase in CKF between the baseline and week 8 assessments (difference = 0.278, 95% CI = 0.014–0.542, *p* = 0.037) (Table 3).

**Table 3:** Between-group differences in muscle strength and pain.

Tests	Treatment group (*n* = 30)	Control group (*n* = 30)	Mean difference (95% CI)	Adjusted *p* value
Symptomatic knee extension muscle strength SKE (N/kg)				
Week 4	2.46 ± 0.81	2.47 ± 0.68	0.01 (−0.335, 0.252)	0.777
Week 8	2.85 ± 0.78	2.45 ± 0.64	0.40 (−0.749,-0.149)	0.004
Symptomatic knee flexion muscle strength SKF (N/kg)				
Week 4	1.87 ± 0.58	1.94 ± 0.57	0.07 (−0.174, 0.339)	0.521
Week 8	2.15 ± 0.55	1.85 ± 0.55	−0.30 (−0.532, −0.032)	0.028
Contralateral knee extension muscle strength CKE (N/kg)				
Week 4	2.68 ± 0.82	2.79 ± 0.81	0.09 (−0.236, 0.418)	0.579
Week 8	3.10 ± 0.82	2.82 ± 0.60	−0.28 (−0.580, −0.033)	0.029
Contralateral knee flexion muscle strength (CKF) (N/kg)				
Week 4	1.95 ± 0.58	1.89 ± 0.50	−0.06 (−0.277, 0.182)	0.679
Week 8	2.18 ± 0.52	1.91 ± 0.59	−0.27 (−0.534, 0.002)	**0.052**
VAS pain (cm)				
Week 4	4.42 ± 1.70	5.18 ± 1.92	0.76 (−1.736, −0.246)	0.010
Week 8	4.15 ± 1.68	4.58 ± 1.62	0.43 (−1.297, −0.013)	0.046

Date expressed as Mean ± SD, baseline data was set as the covariates. One-way ANCOVA was used to determine the group effects. SKE, Symptomatic knee extension muscle strength; SKF, Symptomatic knee flexion muscle strength; CKE, Contralateral knee extension muscle strength; CKF, Contralateral knee flexion muscle strength. VAS, Visual Analogue Scale. The bold value means that the group difference is close to significance (*p* < 0.05).

Notably, no significant time difference was found in the SKE, SKF, CKE, and CKF in the control group.

**Figure 3:**
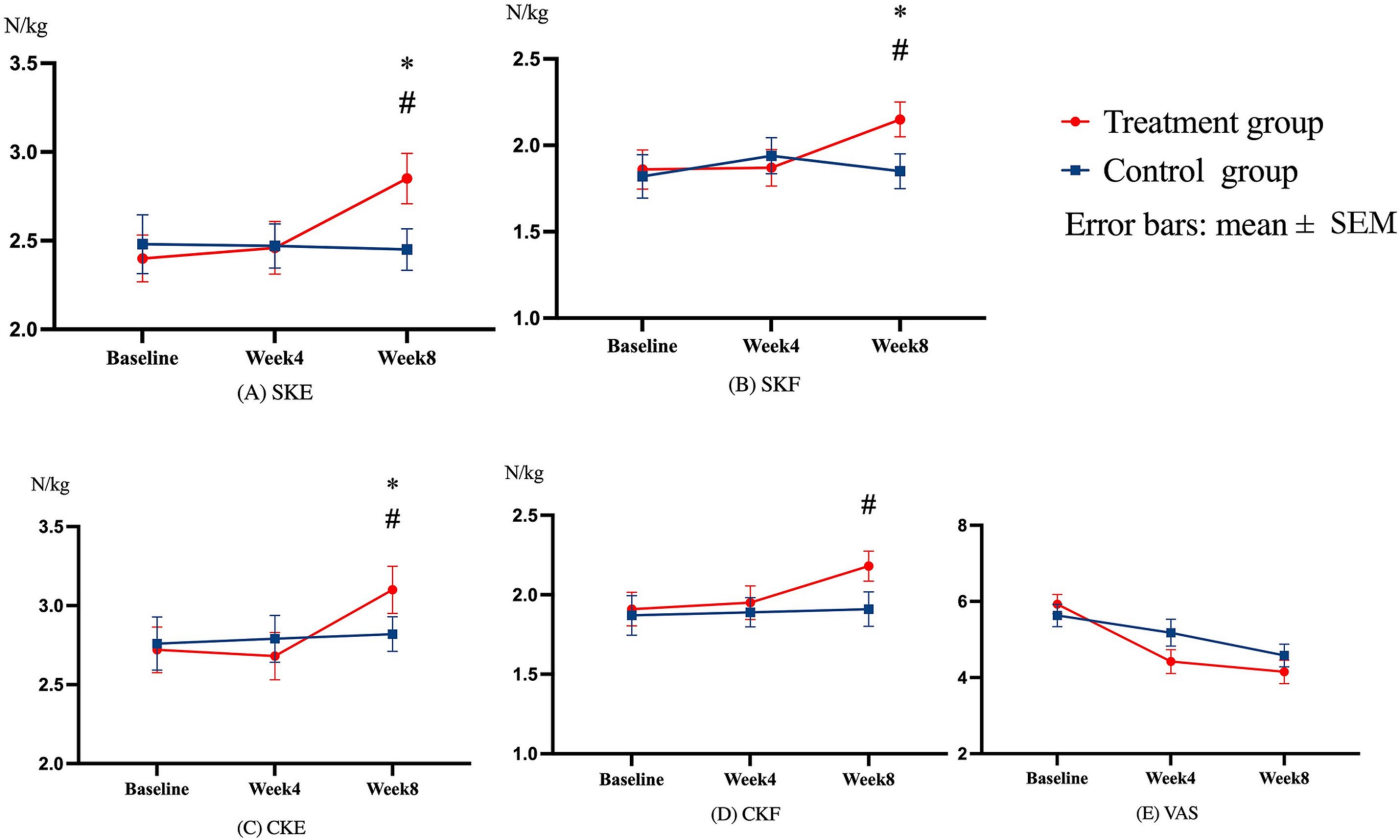
The within-group difference in knee muscle strength and pain A one-way repeated ANOVA was used to compare the within-group difference through **(A–E)**. **(A)** SKE shows the results in symptomatic knee extension muscle strength; **(B)** SKF shows the results in symptomatic knee extension muscle strength; **(C)** CKE shows the results in contralateral knee extension muscle strength; **(D)** CKF shows the results in contralateral knee flexion muscle strength; **(E)** VAS shows Visual Analogue Scale (VAS) pain. Error bars were indicated as the standard deviation (SEM). *Means there was a significant within-group difference (*p* < 0.05) from the baseline to follow-up assessments at week 4 or week 8 in the treatment group. #Means significant within-group difference (*p* < 0.05) from the week 4 to week 8 follow-up assessments. ^Means there was a significant within-group difference (*p* < 0.05) from the baseline to week 8 follow-up assessment in the control group.

A significant time difference was found in the PEMF treatment group in VAS pain [*F*(1.614,46.806) =29.641, *p* < 0.001, partial η^2^ = 0.505]. *Post hoc* analysis of the PEMF treatment group revealed a significant decrease in VAS pain between baseline and week 8 assessments (difference = 1.783, 95% CI = 1.137–2.430, *p* < 0.001) and between baseline and week 4 assessments (difference = 1.517, 95% CI = 0.764–2.269, *p* < 0.001). There was also a significant time difference in the control group in VAS pain [*F*(2, 58) =10.608, *p* < 0.001, partial η^2^ = 0.268]. *Post hoc* analysis revealed a significant decrease in VAS pain between baseline week 8 assessments (difference = 1.050, 95% CI = 0.507–1.593, *p* < 0.001). The compiled data are shown in Figure 3.

### Within-group differences in self-reported and performance-based physical function

Significant time difference was found in gait speed test between baseline and week 8 (difference = 0.051, *p* = 0.026) in the treatment group. No significant time difference was found in the control group.

**Figure 4:**
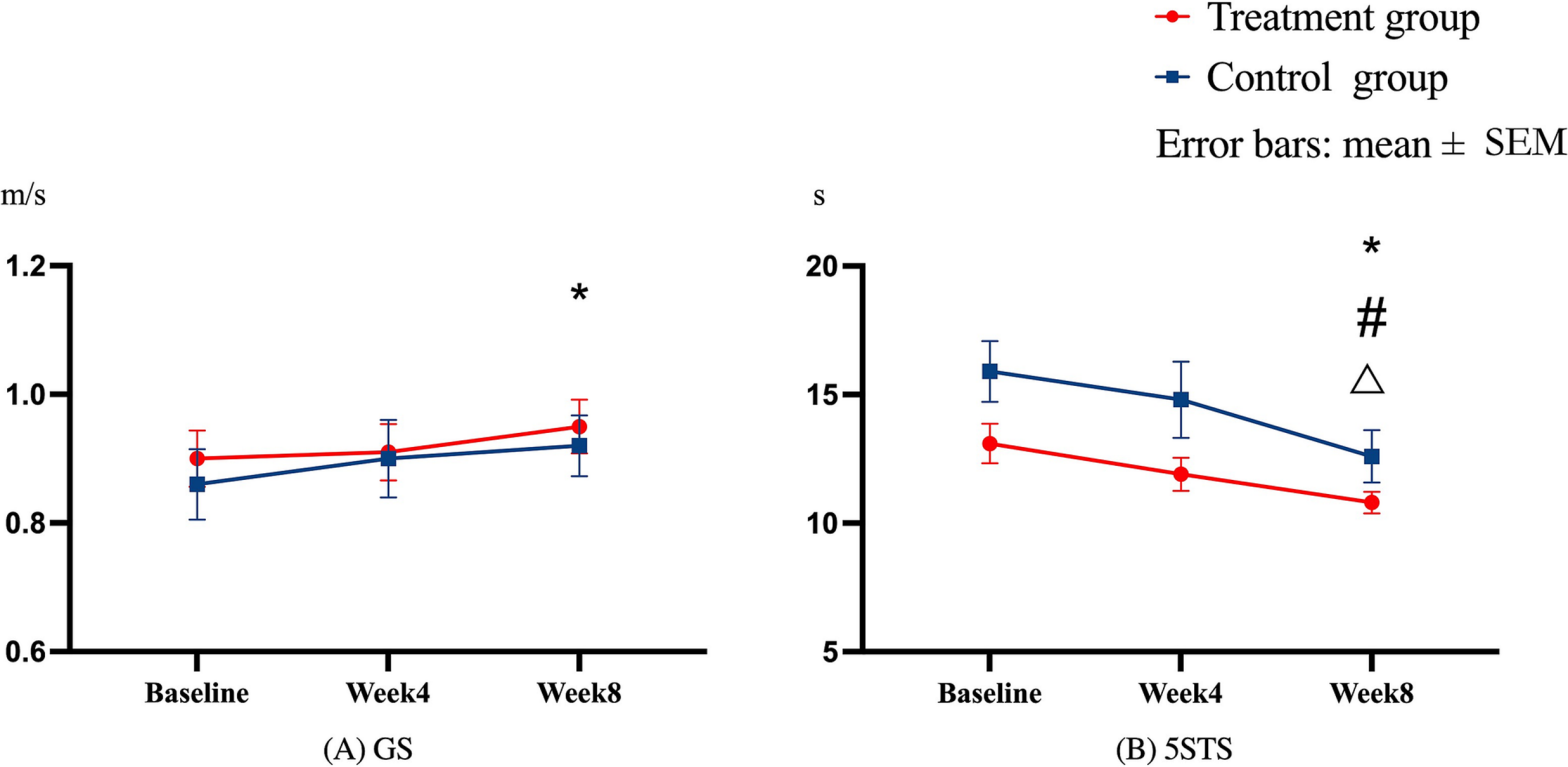
Within-group difference in GS and 5STS A one-way repeated ANOVA was used to compare the within-group difference through **(A,B)**. **(A)** GS, shows the results in gait speed test; **(B)** 5STS shows the results in 5-time sit-to-stand test. Error bars were indicated as the mean ± standard error of the mean (SEM). *Means there was a significant within-group difference (*p* < 0.05) from the Baseline to follow-up assessments at week 4 or week 8 in the treatment group. #Means significant within-group difference (*p* < 0.05) from the week 4 to week 8 follow-up assessments in the treatment group. △There was a significant within-group difference (*p* < 0.05) from the baseline to week 8 follow-up assessment in the control group.

Significant time difference was found in 5-time chair stand test between week 4 and week 8 (difference = 1.164, *p* = 0.030), baseline and week 8 (difference = 2.327, *p* < 0.001) in treatment group. Significant time difference was also found between baseline and week 8 (difference = 3.213, *p* = 0.022) in the control group. The combined results of gait speed test and 5STS test can be seen in the Figure 4. No significant time difference was found in KOOS-ADL.

### Treatment effects (between-group differences) on knee muscle strength and pain

Results from the one-way ANCOVA at the week 8 assessment revealed significant between-group differences in SKE [*F*(1,57) = 8.989, *p* = 0.004, partial η^2^ = 0.136], SKF [*F*(1,57) = 5.094, *p* = 0.028, partial η^2^ = 0.082] and CKE [*F*(1,57) = 5.025, *p* = 0.029, partial η^2^ = 0.081].

There were also significant between-group differences in VAS pain at the week 4 [*F*(1,57) =7.098, *p* = 0.010, partial η^2^ = 0.111] and week 8 assessments [*F*(1,57) =4.186, *p* = 0.046, partial η^2^ = 0.068]. All the results are provided in Table 3.

### Treatment effects (between-group differences) on self-reported and performance-based physical function

No significant group differences were found in the gait speed test, chair-stand test and KOOS-ADL at week 4 and week 8. All the results can be found in Table 4.

**Table 4:** Between-group differences in performance-based and self-reported physical function.

Tests	Treatment group (*n* = 30)	Control group (*n* = 30)	Mean difference (95% CI)	*p* value
Performance-based physical function				
6-meter Gait Speed (m/s)				
Week 4	0.91 ± 0.24	0.90 ± 0.33	−0.01 (−0.079, 0.041)	0.519^b^
Week 8	0.95 ± 0.23	0.91 ± 0.26	0.041 (−0.053, 0.077)	0.717^b^
5-time Chair Stand Test (s)				
Week 4	11.9 ± 3.5	14.8 ± 8.1	2.9 (−0.035, 0.018)	0.694^b^
Week 8	10.8 ± 2.3	12.6 ± 5.6	−1.894 (−2.252, 1.241)	0.497^b^
Self-reported physical function				
KOOS- ADL (score)				
Week 4	70.6 ± 14.6	64.0 ± 19.1	−6.6 (−4.394, 8.155)	0.551^b^
Week 8	70.7 ± 16.4	67.2 ± 17.2	−3.5 (−7.568, 5.874)	0.802^b^

^b One-way ANCOVA was used to determine the group effects with baseline data was set as the covariates.^

### Subgroup analysis

In the subgroup analysis, the significant group difference at week 8 was observed in SKE (*p* = 0.013) in female patients while pain in male patients (*p* = 0.026). Patients aged over 70 years have a significantly superior improvement in SKE, SKF, and CKF after 8 weeks of PEMF therapy. The combined data can be found in the Supplementary material.

### Adverse effects

No adverse effects were reported during treatment and the testing process.

## Discussion

To the best of our knowledge, this is the first study to combine PEMF therapy with home-based exercise in end-stage knee OA. Succinctly, the results demonstrated that 8 weeks of combined intervention of PEMF and home-based exercise led to beneficial changes in knee muscle strength and pain in individuals with end-stage knee osteoarthritis (OA). Notably, both treatment and control groups exhibited significant within-group improvements in performance-based physical function, while no enhancements were observed in self-reported physical function (Figure 4).

### Knee muscle strength

A growing body of evidence shows the importance of exercise in maintaining muscle strength to improve pain (34, 35), physical function (36, 37), and quality of life (37) in knee OA patients. In this study, PEMF therapy significantly increased muscle strength in end-stage knee OA over targeted home-based exercise alone. Several factors may have contributed to the strength improvements observed with PEMF therapy. Firstly, low-energy PEMF therapy (24), similar to exercise (38), stimulates muscular mitochondrial respiration and consequent PGC-1 expression, a known muscle transcriptional cascade (39). Secondly, PEMF therapy does not produce mechanical stress (24). Traditional resistance exercise and, to a lesser degree, endurance exercise, produce mechanical stress and microdamage that, despite spurring muscle remodeling and regeneration, incur a large biosynthetic penalty. On the other hand, muscle mitochondrial activation by brief (10 min) and low energy (1mT at 50 Hz) PEMF exposure does not produce mechanical stress, yet offers a novel way to recapitulate some of the metabolic responses commonly associated with the undertaking of endurance exercise (39).

Although the analgesic attributes of PEMF therapy are accepted and clinically exploited (14, 15, 23), the present study represents a novel application for PEMF therapy with which to recover muscle strength in end-stage knee OA patients. Due to the paucity of detailed clinical guidelines for this technology and the absence of published clinical trials aimed at establishing optimal exposure protocols, with reference to treatment frequency and durations, further investigation of the before mentioned magnetic paradigm is merited. Nonetheless, given the mitohormetic nature of the employed magnetic paradigm (21, 24) caution must also be observed to avoid overexposure and cause undue oxidative stress. As no studies of PEMF mentioned the improvement in knee muscle strength in knee OA patients, we were not able to compare our findings with other researchers’.

### Physical function

In our study, both PEMF therapy plus home-based exercise group (GS & 5STS) and home-based exercise group (5STS) groups showed significant improvements in performance-based physical function, whereby PEMF therapy did not significantly improve effectiveness. This finding is consistent with the findings from another clinical trial that combined PEMF therapy with resistance training for 8 weeks and 24 sessions in performance-based physical function in mild to moderate knee OA patients. Walking speed was improved after 8 weeks of treatment in both the PEMF plus exercise (from 1.08 m/s to 1.67 m/s, mean difference = 0.59) and exercise-only group (1.18 m/s to 1.82 m/s, mean changes = 0.64) but did not have a significant group difference (40). Potential reasons for lack of intervention effect could be the existence of a ceiling effect in the performance-based measures or the possibility that both interventions are equally effective in addressing the functional limitations associated with knee OA, effectively limiting the detection in cumulative improvements and masking time-related changes with no significant group differences. According to one systematic review and meta-analysis, a 30–40% increase in knee-extensor strength is required to be manifested as an improvement in physical function (11). In the present study, as a 20.8% increase was observed in knee extension muscle strength in the treatment group, threshold for physical improvement may not have been achieved. Another possible reason for the lack of effect detection may be the relatively brief 8-week intervention period applied in this study, which may not have been sufficient to produce significant group differences (41). Although knee extensor and flexor muscle strength were associated with performance-based physical function (10). Improvements in muscle strength may take time to translate into functional performance gains as the body may require time to adapt to increased muscle strength in order to effectively incorporate it into complex functional tasks. Significant improvements within the treatment group may have required intervention durations greater than 8 weeks or, alternatively, more frequent exposures per week. Accordingly, functional improvements in gait speed and chair stand tests become more noticeable only after a prolonged period of consistent intervention (42).

Our study did not find any improvement in self-reported physical function. Similarly, Ozguclu and his colleagues did not find the additional effect of 30 min/sessions, 5 sessions/week, 2 weeks of PEMF in self-reported physical function when added to traditional standard care for knee OA patients, which includes hot packs, therapeutic ultrasound, and isometric quadriceps exercises (43). By contrast, Bagnato et al. reported significant improvement in physical function in knee OA patients using wearable PEMF device compared to a placebo group (44). Patients in Bagnato’s study received PEMF treatment for 6 weeks for 12 h a day. In our study, the PEMF paradigm consisted of 10 min/session, 2 sessions/week. Differences in treatment outcome between these studied may be attributed to differences in intervention period, duration of PEMF exposure, or frequency of exposure.

The absence of improvement in self-reported physical function suggests a need for a multifaceted approach to patient education and engagement in therapeutic interventions. Clinicians should consider addressing patient expectations and providing tailored feedback on performance improvements to bridge the gap between objective outcomes and subjective experiences.

The discrepancy between performance-based improvements and self-reported outcomes aligns with previous studies suggesting that objective measures may not always correspond with patients’ perceptions of their functional abilities (45). Performance-based measures can capture the actual ability rather than the presumed capability of a person. This gap may stem from factors such as psychological barriers, expectations, or the chronicity of pain, which can influence self-reported outcomes (46).

### Pain

Most studies examining PEMF therapy for the treatment of knee OA have focused on self-reported pain and physical function. However, several recent systematic reviews have been unable to decisively conclude that PEMF therapy improves symptoms (17, 47, 48). And another review concluded that PEMF therapy could relieve pain, but it was not superior than other conservative therapies, which is inconsistent with our finding (47).The present study found significant group differences in knee pain, consistent with the recent study by Elboim-Gabyzon and Nahhas (25), which showed significant improvements in WOMAC pain rating in early knee OA subjects after receiving twice weekly PEMF sessions (15-min) for three weeks. However, the control group also exhibited a significant, yet smaller, decrease in pain. In the present study, no improvement in muscle strength was observed in the control group, yet pain was improved. Thus, home-based exercise alone (18%) may have had a limited impact on pain measures and PEMF therapy was superior to relieve pain (30%).

### The potential impact of BMI and body weight on study results

There was the significant group difference in body weight and BMI at baseline. Although randomization aims to evenly distribute characteristics like BMI across groups, chance can lead to imbalances (49). So, the first step we have taken is to normalize knee muscle strength (primary outcome), which is a common method to account for differences in body size and set baseline BMI and body weight as covariates to minimize the potential impact.

Weight and BMI are closely linked to physical function in patients with knee osteoarthritis. Higher BMI can exacerbate joint loading and pain, which may influence patients’ performance on functional tests. Many studies have reported that obesity was an important factor in physical function in knee OA patients (50–52). Therefore, differences in baseline weight could contribute to variability in functional outcomes observed between groups, potentially confounding the interpretation of treatment effects.

However, in our previous study, BMI was not associated with physical function in knee OA patients. The reason may be that our BMI in older Asian adults is relatively low compared to what other studies have observed in other ethnicities. In this RCT, the mean BMI is 26.0 in the treatment group and 24.1 in the control group.

#### Age and sex difference

Our results showed that females exhibited greater gains in knee extension strength than males, which is consistent with previous studies (53, 54). Males, on the other hand, experienced greater pain relief following PEMF therapy. The underlying reasons for this gender disparity may be gender-specific psychosocial factors, such as anxiety or depression, which may have influenced treatment outcomes. Women generally exhibit higher levels of anxiety related to chronic pain, which may have affected their response to interventions (55). In this study, patients over 70 years of age were more responsive to PEMF therapy in terms of muscle strength. This could be due to lower muscle strength at baseline in this specific group, which makes gains more pronounced after treatment.

#### Clinical implications

Non-invasive PEMF therapy targets the knee muscles with a specific magnetic signature, which could clinically benefit pain relief and have a promising tendency to improve performance-based physical function after 8-week treatment. The absence of improvement in self-reported physical function suggests a need for a multifaceted approach to patient education and engagement in therapeutic interventions. Clinicians should consider addressing patient expectations and providing tailored feedback on performance improvements to bridge the gap between objective outcomes and subjective experiences.

Early improvements in strength and function can set the stage for better long-term outcomes, potentially delaying the need for surgical interventions, such as total knee replacement. Future researchers could set up the longitudinal studies to investigate if PEMF has the long-term benefits.

### Strengths

Few studies exist that describe non-surgical and non-pharmacological interventions with reported effectiveness in clinical outcomes for patients with severe knee OA. This is the first study to investigate the potential of PEMF therapy to improve muscle strength, physical function, and pain in end-stage knee OA patients. It showed that this conservative treatment could improve such outcomes. Most studies do not report the severity of OA, or focus on KL grades 2 or 3, whereas this study employed end-stage OA patients of KL grades ≥3.

### Limitations

The limitations of the present study are as follows. Firstly, other risk factors associated with knee OA, including nutrition and psychological factors, such as depression, were not assessed or controlled for in the present study. Secondly, this study did not encompass follow-up assessments. Thus, the patient’s long-term compliance or outcomes were not determined. Thirdly, as the muscle-specific PEMF therapy was combined with home-based exercise, evidence to conclude that this PEMF therapy *per se* can improve muscle strength in end-stage knee OA patients is yet to be determined.

## Conclusion

The combination of PEMF therapy and home-based exercise improved knee muscle strength and reduced pain more than home-based exercise alone in subjects with end-stage knee OA and showed promising tendency in improving performance-based physical function. PEMF therapy has been shown to benefit knee muscle strength in female patients and patients aged over 70 years. In addition, male patients were more responsive to PEMF therapy in pain relief. Non-invasive PEMF therapy may represent a safe and convenient adjuvant treatment for end-stage knee OA and merits future clinical studies with longer-term intervention duration and follow-up.

## Data Availability

The raw data supporting the conclusions of this article will be made available by the authors, without undue reservation.
