# Supplementary material for: The effects of pulsed electromagnetic field therapy on muscle strength and pain in patients with end-stage knee osteoarthritis: a randomized controlled trial
Source: Front Med (Lausanne). 2024 Oct 16;11:1435277. doi: 10.3389/fmed.2024.1435277 (PMC11521844; doi:10.3389/fmed.2024.1435277)
Supplement: Supplementary file 1 [file Table_1.DOCX]

**Supplementary table 1. Comparison across different age groups and genders**

|  | **Baseline** | | | **Week4** | | | **Week8** | | |
| --- | --- | --- | --- | --- | --- | --- | --- | --- | --- |
|  | **Treatment group** | **Control**  **group** | **P value**  **(between-group)**  **Independent t-test** | **Treatment group** | **Control group** | **P value**  **(between-group)**  **ANCOVA** | **Treatment group** | **Control group** | **P value**  **(between-group)**  **ANCOVA** |
| Symptomatic knee extension muscle strength (SKE) (N/kg) | | | | | | | | | |
| Gender |  |  |  |  |  |  |  |  |  |
| Male | 2.42 ± 0.84 | 2.86 ± 0.93 | 0.736 | 2.53 ± 0.90 | 2.76 ± 0.87 | 0.930 | 2.84 ± 0.66 | 2.73 ± 0.55 | 0.351 |
| Female | 2.38 ± 0.63 | 2.37 ± 0.90 | 0.450 | 2.41 ± 0.75 | 2.38± 0.61 | 0.893 | 2.87 ± 0.88 | 2.36 ± 0.65 | 0.013 |
| Age |  |  |  |  |  |  |  |  |  |
| 60-70 | 2.54 ± 0.62 | 2.81 ± 0.98 | 0.193 | 2.49 ± 0.81 | 2.49 ± 0.69 | 0.555 | 2.94 ± 0.58 | 2.66 ± 0.57 | 0.131 |
| 70- | 2.25 ± 0.79 | 2.23 ± 0.78 | 0.471 | 2.43 ± 0.73 | 2.45 ± 0.83 | 0.814 | 2.77 ± 0.95 | 2.29 ± 0.65 | 0.021 |
| Symptomatic knee flexion muscle strength (SKF) (N/kg) | | | | | | | | | |
| Gender |  | | | | | | | | |
| Male | 1.96 ± 0.77 | 2.07 ± 1.18 | 0.533 | 2.02 ± 0.75 | 1.91 ± 0.74 | 0.542 | 2.47 ± 0.62 | 1.99 ± 0.62 | **0.054** |
| Female | 1.78 ± 0.49 | 1.74 ± 0.47 | 0.485 | 1.75 ± 0.38 | 1.94 ± 0.53 | 0.168 | 1.90 ± 0.33 | 1.81 ± 0.53 | 0.573 |
| Age |  |  |  |  |  |  |  |  |  |
| 60-70 | 1.95 ± 0.50 | 1.89 ± 0.52 | 0.377 | 1.85 ± 0.61 | 2.18 ± 0.59 | 0.127 | 2.23 ± 0.59 | 2.07 ± 0.45 | 0.467 |
| 70- | 1.77 ± 0.72 | 1.77 ± 0.80 | 0.497 | 1.89 ± 0.57 | 1.75 ± 0.49 | 0.297 | 2.07 ± 0.51 | 1.69 ± 0.57 | 0.018 |
| Contralateral knee extension muscle strength (CKE) (N/kg) | | | | | | | | | |
| Gender |  |  |  |  |  |  |  |  |  |
| Male | 2.73 ± 0.84 | 2.89 ± 0.97 | 0.370 | 2.67 ± 0.86 | 2.93 ± 0.90 | 0.608 | 3.17 ± 0.85 | 2.96 ± 0.44 | 0.298 |
| Female | 2.70 ± 0.78 | 2.72 ± 0.92 | 0.632 | 2.69 ± 0.81 | 2.75 ± 0.80 | 0.792 | 3.05 ± 0.83 | 2.78 ± 0.63 | 0.098 |
| Age |  |  |  |  |  |  |  |  |  |
| 60-70 | 2.92 ± 0.61 | 3.23 ± 0.83 | 0.131 | 2.77 ± 0.90 | 3.09 ± 0.86 | 0.755 | 3.18 ± 0.74 | 3.15 ± 0.54 | 0.422 |
| 70- | 2.52 ± 0.92 | 2.39 ± 0.84 | 0.345 | 2.59 ± 0.75 | 2.57 ± 0.71 | 0.829 | 3.03 ± 0.92 | 2.57 ± 0.51 | 0.043 |
| Contralateral knee flexion muscle strength (CKF) (N/kg) | | | | | | | | | |
| Gender |  |  |  |  |  |  |  |  |  |
| Male | 1.95 ± 0.74 | 2.02 ± 0.96 | 0.439 | 2.08 ± 0.78 | 2.00 ± 0.70 | 0.630 | 2.41 ± 0.61 | 2.20 ± 0.70 | 0.421 |
| Female | 1.87 ± 0.45 | 1.82 ± 0.60 | 0.328 | 1.85 ± 0.36 | 1.85 ± 0.43 | 0.929 | 2.01 ± 0.38 | 1.82 ± 0.54 | 0.217 |
| Age |  |  |  |  |  |  |  |  |  |
| 60-70 | 2.03 ± 0.49 | 2.06 ± 0.59 | 0.450 | 1.98 ± 0.62 | 2.04 ± 0.48 | 0.819 | 2.27 ± 0.50 | 2.10 ± 0.49 | 0.388 |
| 70- | 1.78 ± 0.66 | 1.72 ± 0.73 | 0.416 | 1.92 ± 0.57 | 1.77 ± 0.49 | 0.350 | 2.10 ± 0.54 | 1.75 ± 0.63 | 0.095 |
| 6-meter gait speed (m/s) | | | | | | | | | |
| Gender |  |  |  |  |  |  |  |  |  |
| Male | 0.94 ± 0.26 | 1.06 ± 0.31 | 0.425 | 0.99 ± 0.26 | 1.20 ± 0.32 | 0.080 | 1.02 ± 0.26 | 1.15 ± 0.30 | 0.680 |
| Female | 0.86 ± 0.23 | 0.80 ± 0.28 | 0.546 | 0.85 ± 0.21 | 0.80 ± 0.28 | 0.727 | 0.89 ± 0.20 | 0.83 ± 0.21 | 0.628 |
| Age |  |  |  |  |  |  |  |  |  |
| 60-70 | 0.95 ± 0.23 | 0.96 ± 0.22 | 0.430 | 0.96 ± 0.22 | 0.97 ± 0.25 | 0.444 | 1.00 ± 0.22 | 0.95 ± 0.19 | 0.099 |
| 70- | 0.85 ± 0.25 | 0.78 ± 0.34 | 0.271 | 0.84 ± 0.24 | 0.84 ± 0.38 | 0.126 | 0.89 ± 0.24 | 0.87 ± 0.31 | 0.497 |
| 5-time Chair Stand Test (s) | | | | | | | | | |
| Gender |  |  |  |  |  |  |  |  |  |
| Male | 12.2 ± 3.30 | 11.8 ± 5.73 | 0.271 | 11.3 ± 1.96 | 11.2 ± 5.45 | 0.714 | 10.6 ± 1.98 | 9.8 ± 1.98 | 0.439 |
| Female | 13.8 ± 4.83 | 17.1 ± 6.35 | 0.480 | 12.4 ± 4.30 | 15.9 ± 8.54 | 0.813 | 10.9 ± 2.52 | 13.5 ± 5.66 | 0.332 |
| Age |  |  |  |  |  |  |  |  |  |
| 60-70 | 12.2 ± 4.17 | 14.6 ± 6.48 | 0.118 | 10.6 ± 2.07 | 13.2 ± 6.77 | 0.525 | 9.99 ± 2.00 | 11.1 ± 4.60 | 0.966 |
| 70- | 14.1 ± 4.21 | 16.8 ± 6.59 | 0.093 | 13.3 ± 4.14 | 16.1 ± 8.97 | 0.985 | 11.6 ± 2.30 | 13.8 ± 6.04 | 0.468 |
| VAS pain (cm) | | | | | | | | | |
| Gender |  | | | | | | | | |
| Male | 6.15 ± 1.41 | 5.86 ± 1.21 | 0.763 | 4.54 ± 1.46 | 5.00 ± 1.83 | 0.287 | 4.23 ± 1.82 | 5.28 ± 1.50 | 0.026 |
| Female | 5.76 ± 1.40 | 5.57 ± 1.72 | 0.510 | 4.32 ± 1.90 | 5.24 ± 1.98 | 0.036 | 4.09 ± 1.61 | 4.37 ± 1.63 | 0.315 |
| Age |  |  |  |  |  |  |  |  |  |
| 60-70 | 5.90 ± 1.63 | 5.27 ± 1.98 | 0.181 | 4.63 ± 2.01 | 5.04 ± 2.47 | 0.129 | 4.17 ± 2.01 | 4.23 ± 1.63 | 0.274 |
| 70- | 5.97 ± 1.17 | 5.91 ± 1.24 | 0.449 | 4.20 ± 1.21 | 5.29 ± 1.43 | 0.016 | 4.13 ± 1.34 | 4.85 ± 1.62 | 0.103 |
| KOOS- ADL (score) | | | | | | | | | |
| Gender |  |  |  |  |  |  |  |  |  |
| Male | 73.5 ± 13.3 | 73.9 ± 17.0 | 0.182 | 72.3 ± 16.5 | 76.3 ± 16.5 | 0.574 | 69.0 ± 18.9 | 75.4 ± 13.7 | 0.400 |
| Female | 69.5 ± 12.4 | 62.8 ± 17.0 | 0.105 | 69.4 ± 13.3 | 60.3 ± 18.6 | 0.331 | 71.9 ± 14.7 | 64.7 ± 17.7 | 0.612 |
| Age |  |  |  |  |  |  |  |  |  |
| 60-70 | 68.5 ± 11.1 | 72.9 ± 16.5 | 0.205 | 71.3 ± 10.5 | 66.8 ± 18.9 | 0.017 | 72.7 ± 13.5 | 73.8 ± 15.6 | 0.599 |
| 70- | 73.9 ± 14.0 | 59.6 ± 16.1 | 0.006 | 69.9 ± 18.2 | 61.9 ± 19.6 | 0.434 | 68.7 ± 19.2 | 62.2 ± 17.1 | 0.486 |
